# The Human Polyomavirus Middle and Alternative T-Antigens; Thoughts on Roles and Relevance to Cancer

**DOI:** 10.3389/fmicb.2018.00398

**Published:** 2018-03-08

**Authors:** Els van der Meijden, Mariet Feltkamp

**Affiliations:** Department of Medical Microbiology, Leiden University Medical Center, Leiden, Netherlands

**Keywords:** polyomavirus, T-antigens, oncogenesis, cell-signaling, evolution

## Abstract

Approximately 15–20% of human cancer is related to infection, which renders them potentially preventable by antimicrobial or antiviral therapy. Human polyomaviruses (PyVs) are relevant in this regard, as illustrated by the involvement of Merkel cell polyomavirus (MCPyV) in the development of Merkel cell carcinoma. The polyomavirus Small and Large tumor antigen (ST and LT) have been extensively studied with respect to their role in oncogenesis. Recently it was shown that a number of human PyVs, including MCPyV and the trichodysplasia spinulosa polyomavirus (TSPyV), express additional T-antigens called Middle T (MT) and alternative T (ALT). ALT is encoded by ORF5, also known as the alternative T open reading frame (ALTO), which also encodes the second exon of MT, and overlaps out-of-frame with the second exon of LT. Previously, MT was considered unique for oncogenic rodent polyomaviruses, and ALT was still unknown. In this mini-review, we want to point out there are important reasons to explore the involvement of MT and ALT in human cellular transformation. First, just like their rodent equivalents, MT and ALT probably disrupt cellular pathways that control signaling and proliferation. Second, expression of the MT and ALT-encoding ORF5/ALTO characterizes a monophyletic polyomavirus clade that includes human and animal PyVs with known oncogenic potential. And third, ORF5/ALTO is subject to strong positive selection aimed specifically at a short linear motif within MT and ALT that overlaps completely with the RB-binding motif in LT. The latter suggests tight interplay between these T-antigens with possible consequences for cell transformation.

## Background

An established causal relationship between a certain cancer and a particular infection provides ample opportunities to study oncogenesis in detail, and identify unique targets for cancer prevention and treatment. The best example in this regard is probably cervical cancer and human papillomavirus (HPV) infection. The E6 and E7 oncoproteins encoded by high risk HPV types are directly involved in tumorigenesis and maintenance of the transformed state (McLaughlin-Drubin and Munger, 2009). Such knowledge has contributed directly to development of preventive HPV vaccines that reduce the incidence of HPV-related (pre)malignancies (Harper and DeMars, 2017).

The rapidly expanding group of human polyomaviruses (PyVs) is also relevant in this regard. For one human PyV, the Merkel cell polyomavirus (MCPyV), causal involvement in the development of Merkel cell carcinoma (MCC) has been established (Feng et al., 2008; Shuda et al., 2008; Houben et al., 2010). Consequently, MCPyV has been defined as a group 2A carcinogenic biological agent (Bouvard et al., 2012). The polyomavirus Small and Large T-antigens (ST and LT) that resemble HPV E6 and E7 in several ways, are the usual suspects when it comes to cellular transformation, as they are known to deregulate cellular pathways controlling the cell cycle, DNA repair, and apoptosis. The focus of this mini-review, however, will be on two other T-antigens, Middle T (MT) and Alternative T (ALT), both encoded by the Alternate T-antigen open reading frame (ALTO), also known as ORF5, depending on the polyomavirus in which the open reading frame was recognized (Carter et al., 2013; van der Meijden et al., 2013b; Lauber et al., 2015). ORF5/ALTO is expressed by MCPyV (Carter et al., 2013) and by the trichodysplasia spinulosa polyomavirus (TSPyV) that causes dysplasia of human hair inner root sheath cells and follicular spine formation of the skin (van der Meijden et al., 2010, 2015). While for MCPyV ALTO only ALT expression has been demonstrated, TSPyV ORF5 was shown to express both MT and ALT. This mini-review will address what is currently known about the human PyV MT and ALT products and speculate about their role in human cellular transformation.

## The Polyomavirus T-Antigens, Especially the ORF5/ALTO-Encoded MT and ALT Products

Because the human PyVs cause rare diseases observed only in specific immunocompromised patient groups, they are less known. Nevertheless they have striking similarities with HPV, including inactivation of the tumorsuppressor proteins p53 and RB through the T-antigens (DeCaprio and Garcea, 2013). Until recently human PyVs were believed to express only two major T-antigens, ST and LT, next to some smaller splice products thereof (Gjoerup and Chang, 2010). Rodent PyVs, including some oncogenic ones (Gross, 1953; Eddy et al., 1962; Girardi et al., 1962), are known to express an additional T-antigen called MT (Ito et al., 1977; Courtneidge et al., 1991; Fluck and Schaffhausen, 2009; Schaffhausen and Roberts, 2009).

Until the discovery of MCPyV in 2008, the T-antigens were merely seen as viral oncoproteins useful to study oncogenesis *in vitro* and in animal models, for instance the murine polyomavirus (MPyV) in the mouse mammary tumor virus (MMTV) breast cancer model (Fluck and Schaffhausen, 2009). This situation changed considerably with the identification of MCPyV in human MCCs (Feng et al., 2008). MCC is a rare but aggressive skin tumor of neuroendocrine origin, although early B-cells have also been proposed as cells of origin (Sauer et al., 2017). The majority of MCCs harbor clonally integrated MCPyV genome copies that express ST and preliminary truncated versions of LT (Feng et al., 2008; Shuda et al., 2008, 2009; Houben et al., 2010). Studies have shown that MCPyV-positive MCC tumor cell growth depends on the interaction of LT with RB (Houben et al., 2012), while ST promotes cell proliferation by deregulation of the mTOR signaling pathway via inactivation of 4E-BP1 (Shuda et al., 2011; Velasquez et al., 2016). The contribution of ALT and MT to development of MCC or any other human tumor type, respectively, is not known at the moment. Below, we summarize some recent findings that should prompt the interest in MT and ALT as potential viral oncoproteins that merit further study.

## Oncogenic Polyomaviruses Expressing ORF5/ALTO Phylogenetically Cluster Together

**FIGURE 1:**
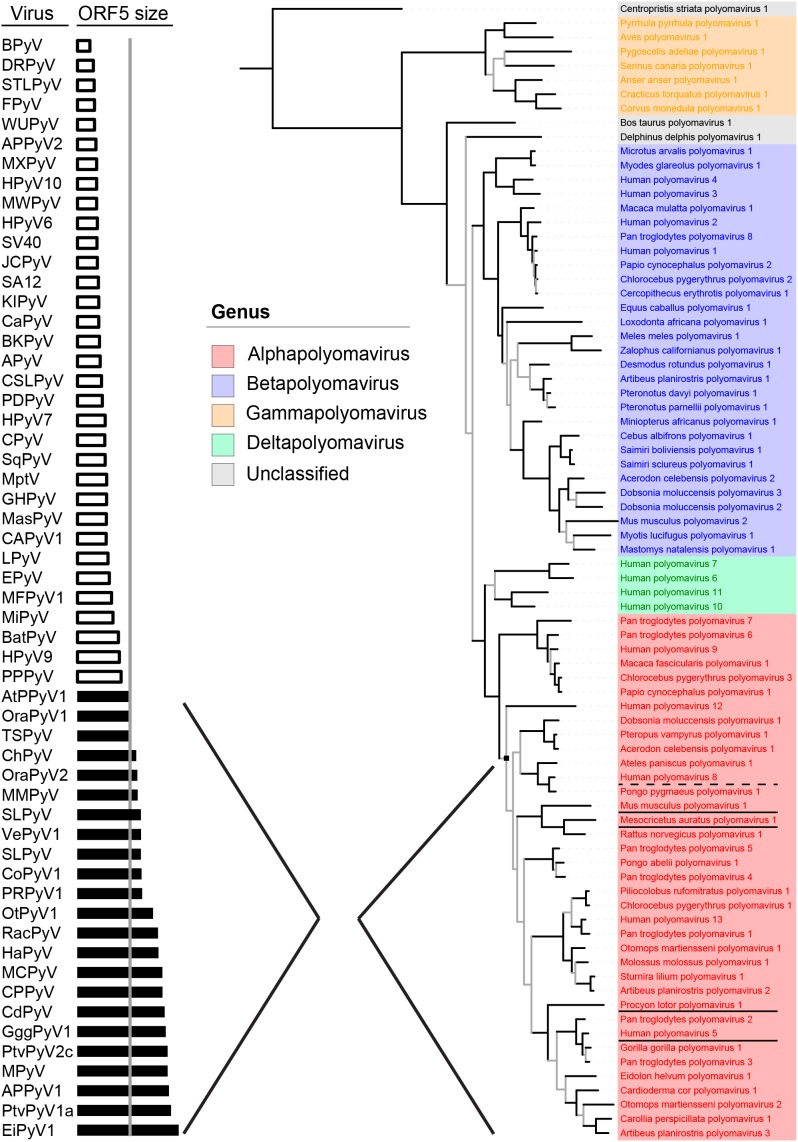
Evolutionary conservation of Middle T and ALT (ORF5) Bayesian phylogeny based on conserved regions in LT, as used by the Study Group of the ICTV for the demarcation of genera (adapted from Moens et al., 2017b) is shown on the right. On the left, polyomaviruses are ordered by ORF5 size. Shown in black bars are intact versions of ORF5, containing the characteristic disordered domains, SLIMs, proline-rich areas and a C-terminal transmembrane domain, that define the major clade (node) of the *alphapolyomaviruses* shown in red on the right. *Alphapolyomaviruses* with known oncogenic or dysplastic properties are respectively underlined or with dashed line.

Recently, the Polyomaviridae Study Group of the International Committee on Taxonomy of Viruses (ICTV) (Polyomaviridae Study Group of the International Committee on Taxonomy of Viruses et al., 2016; Moens et al., 2017a) established a new phylogeny-based taxonomy based on conserved regions in LT. This resulted in the demarcation of four genera (*alpha*–*delta*) within the polyomavirus family (**Figure 1**, right part), and the recognition of 13 human PyV *species* found among three genera (*alpha*, *beta*, and *delta)*. The *alpha* genus contains many (if not all) known naturally oncogenic PyVs, underlined in **Figure 1**, including MCPyV (*Human polyomavirus 5*), the raccoon polyomavirus (RacPyV, *Procyon lotor polyomavirus 1*) (Dela Cruz et al., 2013), MPyV (*Mus musculus polyomavirus 1*) and hamster PyV (*Mesocricetus auratus polyomavirus 1*) (Gross, 1953; Graffi et al., 1969). The dysplasia-inducing TSPyV (*Human polyomavirus 8*) also belongs to the *alpha* genus (van der Meijden et al., 2010; Kazem et al., 2012). The BK and JC polyomaviruses (*Human polyomavirus 1 and 2*), which are sometimes implied in bladder and colon cancer, respectively (Dalianis and Hirsch, 2013), do not belong to the alpha genus (**Figure 1**, in blue).

**FIGURE 2:**
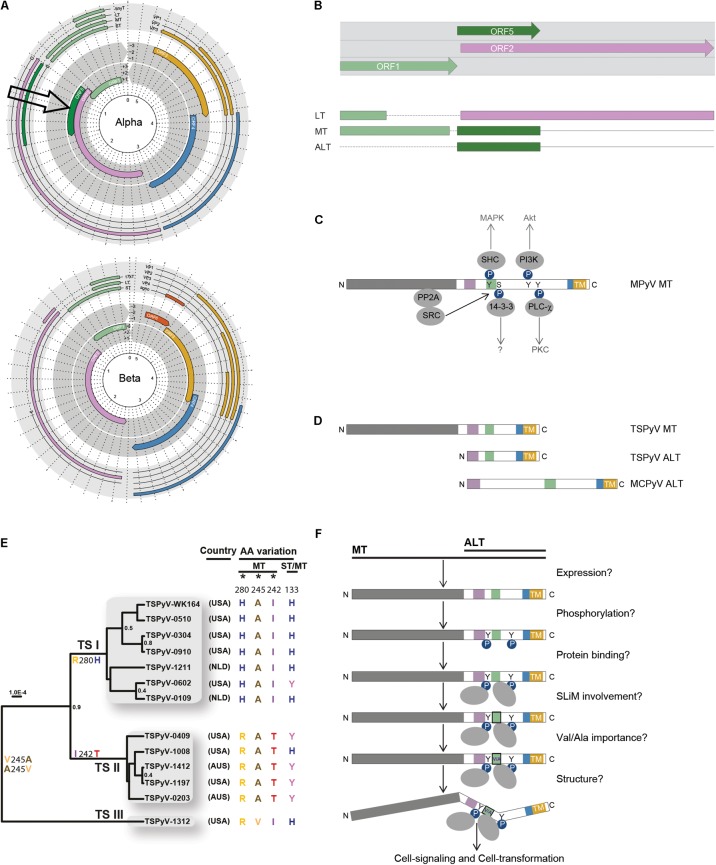
Expression, evolution, and putative role of ORF5 and MT/ALT in cell-signaling and transformation. **(A)** Genomes of an *alpha* and *betapolyomavirus* are shown (adapted from Moens et al., 2017b). Notice the presence of ORF5 in the *alphapolyomavirus* genome (arrow). **(B)** ORF5 encodes the second exon of MT and ALT, and overlaps with the second exon of LT. The colors of the (spliced) T-antigens correspond to the encoding ORF. **(C)** Phosphorylation of mouse polyomavirus (MPyV) MT by Src tyrosine kinase interacting with PP2A-bound by the N-terminal part of MT shown in gray. Phosphorylated MT induces docking and activation of members of the signal transduction pathway, SHK, PI3K, 14-3-3, and PLC (Dilworth, 2002). The colored blocks represent SLiMs present in the intrinsically disordered region (IRD) shown in white (Lauber et al., 2015). The C-terminal transmembrane domain (TM) is shown in yellow. Phosphorylation (P-spheres) of tyrosines (Y) and protein interactions (gray ovals) are depicted. **(D)** Schematic representation of human MT and ALT expressed by TSPyV and MCPyV (colors and shapes as explained in **C**). **(E)** TSPyV phylogeny adapted with permission from Kazem et al. (2016) showing three lineages of TSPyV defined by predominant non-synonymous substitutions in MT/ALT during evolution. **(F)** Schematic overview of HPyV MT/ALT related issues regarding its state, structure and role (colors and shapes as explained in **C**).

As far as reported, *alpha* PyVs, including three additional human PyVs so far without attributable disease (HPyV9, HPyV12, and New Jersey PyV), are prevalent in the general population, with a seroprevalence up to 80% (Kean et al., 2009; Nicol et al., 2013; van der Meijden et al., 2013a; Gossai et al., 2016). In our laboratory and elsewhere it was shown that only *alphapolyomaviruses* contain a full length ORF5/ALTO open reading frame (Carter et al., 2013; Lauber et al., 2015; van der Meijden et al., 2015; Moens et al., 2017b) (**Figures 1**, **2A**, arrow). The ORF5/ALTO-like shorter open reading frame in PyVs from other genera contains a premature stop codon and does not encode the hydrophobic C-terminus. Full length ORF5/ALTO was previously recognized only in the (oncogenic) hamster and murine PyVs. It is also found in RacPyV causing brain tumors in raccoons (Brostoff et al., 2014).

## Alternative Expression of ORF5/ALTO Involves LT Transcription

The location and context of ORF5/ALTO in the PyV genome is shown in **Figures 2A,B**. ORF5/ALTO overlaps entirely (out of frame) with the second exon of LT, which is encoded by ORF2. As shown by us for TSPyV (van der Meijden et al., 2015), ORF5/ALTO is expressed in two different ways to encode MT or ALT. MT is expressed through alternative splicing resulting in a transcript that combines exon 1, which largely overlaps with ST and the first exon of LT, with exon 2 encoded by ORF5/ALTO. Next to this regular expression pattern seen in many more PyVs and HPVs, ORF5/ALTO can be expressed also on its own to encode ALT. For this purpose, the major T-transcript encoding LT is used (van der Meijden et al., 2015). *In vitro* at least, the latter route seems to generate the most abundant product, as much more ALT than MT protein is detected (van der Meijden et al., 2015). For MCPyV thus far only ALT expression from ORF5/ALTO was shown. How the major start codon used by the LT transcript is avoided and whether ALT expression involves an internal ribosome entry site (IRES) and the use of a downstream alternative start codon has not been resolved yet.

It is challenging to speculate why ORF5/ALTO is expressed in different ways, from different transcripts. We are not aware of any other viral product that is encoded like this. Possibly ORF5/ALTO expression, either as MT or as ALT, is of crucial importance to the virus life cycle and therefore needs to be ensured at all times, for example during different stages of the host cell cycle or in differentiating host cells. Alternatively, alternating ALTO expression might play a role in regulating LT expression, as there might be competition between ALT and LT for the same transcript. Further studies are needed to explore this possible regulatory process, which might impact on the transforming potential of LT as well. In the case of MCPyV, there might be a correlation with the truncation of MCPyV LT observed in MCC cells, which might call for ALT expression, for instance to supplement a necessary factor normally provided by LT. It should be noticed, however, that in MCC also ALT is often truncated (Carter et al., 2013). Whether the capacity to express both MT and ALT (e.g., TSPyV) opposed to only ALT (e.g., MCPyV) correlates with the capacity to integrate in the host genome as shown for MCPyV is unknown at the moment.

## The ORF5/ALTO Encoded MT and ALT Products Are Largely Intrinsically Disordered and Likely Involved in Cell-Signaling

The shared C-terminal part of MT and ALT encoded by ORF5/ALTO contains several small conserved regions called short linear motifs (SLiMs, colored blocks in **Figures 2C,D**) within larger intrinsically disordered regions (IDRs) (Lauber et al., 2015). IDR and SLiM-containing unstructured proteins are frequently encoded by overlapping ORFs and involved in numerous biologic processes related to disease, including cancer (van der Lee et al., 2014; Xue et al., 2014). As expected for intrinsically disordered proteins, comparison of the shared MT and ALT encoding sequences between different *alphapolyomaviruses* reveals little homology.

From murine studies we know that the second part of MT is proline-rich, contains phosphotyrosine and phosphoserine residues and harbors a transmembrane domain at the very C-terminal end (**Figure 2C**) (Dilworth, 2002; Cheng et al., 2009; Fluck and Schaffhausen, 2009). Functionally, MT acts as an engaged transmembrane growth factor receptor (Dilworth, 2002; Fluck and Schaffhausen, 2009). Upon membrane and PP2A binding, MT interacts with Src tyrosine kinase, which results in phosphorylation of MT and subsequent activation of important phosphokinases, such as PI3K and MAPK involved in cell-signaling (**Figure 2C**) (Dilworth, 2002). Furthermore, the Hippo pathway effectors Taz and Yap, bind to MPyV MT and thereby support Src activation and cellular transformation by MT (Rouleau et al., 2016; Shanzer et al., 2017).

Expression of a full-length ORF5/ALTO (**Figure 1**, black bars), containing the characteristic disordered domains, the SLIMs, the proline-rich areas, the phosphoamino acid residues and the transmembrane domain, defines the vast majority of PyVs within the *alpha* genus (**Figure 1**), including MCPyV and TSPyV. Whether human PyV MT and ALT also show established rodent MT-like properties and are potentially involved in cellular transformation is not known yet. From sequence comparisons showing that the involved motifs are present, for example PP2A in MT, this seems likely. In co-immunoprecipitation experiments and with mutational analysis it was shown that TSPyV MT interacts with PP2A, which involves activation of the MAPK signaling pathway (Wu et al., 2017), as described for MPyV MT (Dilworth, 2002; Cheng et al., 2009; Fluck and Schaffhausen, 2009). We have recently obtained preliminary data showing that MCPyV ALT indeed is phosphorylated.

For MCPyV it was shown that an intact transmembrane domain is required for the subcellular distribution pattern of ALT, comparable to discrete subcellular membrane binding observed for MPyV MT (Zhou et al., 2011; Carter et al., 2013). MPyV MT mutant studies showed that membrane association is required for MT-induced transformation (Novak and Griffin, 1981; Carmichael et al., 1982).

## Evolutionary Conservation of ORF5/ALTO Is Tightly Linked to the LT RB-Binding MOTIF

As illustrated above, ORF5/ALTO expression seems important for specific human PyVs, including MCPyV and TSPyV. This relevance is underscored by two recent studies that looked into the evolutionary conservation of ORF5/ALTO among PyVs, especially members of the *alpha* genus (Carter et al., 2013; Lauber et al., 2015). Especially the latter study showed that expression of a full-length ORF5/ALTO (**Figure 1**, black bars), containing the characteristic disordered domains, the SLIMs, the proline-rich areas and the transmembrane domain, defines the vast majority of PyVs within the *alpha* genus (**Figure 1**).

Single nucleotide polymorphism and evolutionary analyses of TSPyV revealed that ORF5/ALTO is the predominant site of non-synonymous substitutions during PyV evolution (**Figure 2E**) (Kazem et al., 2016), subject to strong positive selection (Lauber et al., 2015). Analysis of the entire PyV family showed this pressure is focused on a single amino acid position (MT245/ALT44 for TSPyV, **Figure 2E**) located within a conserved SLiM (green block in **Figures 2C,D**) allowing only two SLiM variants. The latter is highly unusual among conserved residues and results in binary toggling between Valine and Alanine. Particular interesting in this regard is the information that this restriction is imposed by the highly conserved RB-binding motif (LXCXE) within LT, which completely overlaps with the relevant SLiM (Lauber et al., 2015).

The impact of this finding might seem trivial, since no preference for either a Valine or an Alanine residue is observed among oncogenic PyVs or on the (human) *alphapolyomaviruses* as a whole. However, a dynamic analysis among PyV species taking into account the binary exchange rate of the second codon position corresponding to either Valine–Alanine clearly showed that codon-constrained Valine–Alanine (COCO-VA) toggling is significantly accelerated in ORF5-containing (*alpha)polyomaviruses* compared to ORF5-less viruses (Lauber et al., 2015).

Comparison of 13 TSPyV genomes showed one divergent isolate with a Valine instead of an Alanine residue at position 245 (TSPyV-1312, **Figure 2E**) (Kazem et al., 2016). Adaptation within one species is not observed for any other PyV. For instance in MCPyV, an Alanine residue at position 245 is present in all GenBank-deposited genome isolates regardless of expression of full-length or truncated ALT.

The finding of two overlapping, extremely conserved motifs (the MT/ALT SLiM and the LT RB-binding motif), again suggests that the functional roles of MT/ALT and LT are mutually connected. Since the highly conserved RB-binding motif is also present in the E7 gene of HPV (McLaughlin-Drubin and Munger, 2009), and in the E1A gene of adenoviruses (Munger et al., 2004), we looked for a comparable situation in these related virus families. However, in both of these virus families the RB-binding motif is located in a non-overlapping coding region of the genome, highlighting the unique constellation of the ORF5/ALTO-expressing PyVs.

In general, the presence of two overlapping conserved motifs is rare. More often the organization of functional motifs is such that critical functional residues in one protein overlap with highly mutable regions of the other protein, like observed in the overlapping E2 and E4 genes, and the tat and rev genes of HPV and HIV1, respectively (Hughes and Hughes, 2005; Narechania et al., 2005; Fernandes et al., 2016).

## Conclusion, Discussion, and Perspective

So far, searches into the role of polyomaviruses in human cancer have focused on the traditional T-antigens LT and ST. Since expression of full length ORF5/ALTO characterizes members of the ‘oncogenic’ *alphapolyomavirus* genus, we consider it important to study the transforming properties of MT and ALT as well. Next to straightforward approaches investigating involvement of MT and ALT in human cell-signaling and cell cycle regulation through phosphorylation (**Figure 2F**), the relevance of the identified MT/ALT SLiM and Valine–Alanine variants on cell-signaling and cell cycle regulation merits further study. Since SLiMs are involved in (transient) protein interactions and folding of disordered proteins (Davey et al., 2012) (COCO-VA) SLiM variants might impact on the structure, protein-binding and role of MT and ALT (**Figure 2F**). These investigations should take into account the role of the overlapping, possibly competing or supplementing, LT product with known transforming properties that contains the MT/ALT SLiM-overlapping RB-binding motif. Altogether, we believe studying MT and ALT in conjunction with LT will provide valuable insight in the biology of PyV, which will further reveal the involvement of PyV infection in human cancer and possibly provide new targets for cancer prevention and treatment.

## Author Contributions

All authors listed have made a substantial, direct and intellectual contribution to the work, and approved it for publication.

## Conflict of Interest Statement

The authors declare that the research was conducted in the absence of any commercial or financial relationships that could be construed as a potential conflict of interest. The handling Editor declared a past co-authorship with one of the authors MF.
